# Genotyping of a microsatellite locus to differentiate clinical Ostreid herpesvirus 1 specimens

**DOI:** 10.1186/1297-9716-45-3

**Published:** 2014-01-10

**Authors:** Tristan Renault, Gwenaëlle Tchaleu, Nicole Faury, Pierrick Moreau, Amélie Segarra, Valérie Barbosa-Solomieu, Sylvie Lapègue

**Affiliations:** 1 Ifremer, Unite Santé, Génétique et Microbiologie des Mollusques, Laboratoire de Génétique et Pathologie des Mollusques Marins, 17390 La Tremblade, France

## Abstract

Ostreid herpesvirus 1 (OsHV-1) is a DNA virus belonging to the *Malacoherpesviridae* family from the Herpesvirales order. OsHV-1 has been associated with mortality outbreaks in different bivalve species including the Pacific cupped oyster, *Crassostrea gigas*. Since 2008, massive mortality events have been reported among *C. gigas* in Europe in relation to the detection of a variant of OsHV-1, called μVar. Since 2009, this variant has been mainly detected in France. These results raise questions about the emergence and the virulence of this variant. The search for association between specific virus genetic markers and clinical symptoms is of great interest and the characterization of the genetic variability of OsHV-1 specimens is an area of growing interest. Determination of nucleotide sequences of PCR-amplified virus DNA fragments has already been used to characterize OsHV-1 specimens and virus variants have thus been described. However, the virus DNA sequencing approach is time-consuming in the high-scale format. Identification and genotyping of highly polymorphic microsatellite loci appear as a suitable approach. The main objective of the present study was the development of a genotyping method in order to characterise clinical OsHV-1 specimens by targeting a particular microsatellite locus located in the ORF4 area. Genotyping results were compared to sequences already available. An excellent correlation was found between the detected genotypes and the corresponding sequences showing that the genotyping approach allowed an accuraté discrimination between virus specimens.

## Introduction

Ostreid herpesvirus 1 (OsHV-1) is a DNA virus belonging to the *Malacoherpesviridae* family from the Herpesvirales order [1]. The virus has been purified from naturally infected *Crassostrea gigas* larvae [2] and its genome entirely sequenced [3]. The viral genome is a large linear duplex DNA molecule of 207 kb (GenBank accession number AY509253) that encodes at least 124 genes [3].

OsHV-1 has been associated with mortality outbreaks in different bivalve species including the Pacific cupped oyster, *C. gigas*[4,5]. Since 2008, massive mortality outbreaks have been reported among *C. gigas* spat in several farming areas in Europe [6-11] in relation to the detection of a newly described OsHV-1 variant called μVar [7]. Although the reference type (a viral specimen collected in France in 1995 during a mortality event affecting *C. gigas* larvae, GenBank accession number AY509253) and the variant μVar were detected in association with mortality outbreaks in 2008 in France, virus detection since 2009 has mainly concerned the μVar variant [7,10,12]. These results raise questions about the emergence and the virulence of the μVar variant. In this context, tools are needed in order to better describe OsHV-1 diversity in relation to virulence and geographical distribution. In light of the genetic diversity of OsHV-1, the search for associations between specific virus genetic markers and clinical symptoms is of great interest.

Determination of nucleotide sequences of PCR-amplified virus DNA fragments is the most accurate method for virus genotyping [13]. The DNA sequencing approach has been used to characterise OsHV-1 specimens and virus variants were thus reported [7,10,12,14-19]. The μVar variant [7] showed several differences in two genome areas when compared with the reference type (GenBank accession n° AY509523) and all these differences need to be observed to define a viral specimen as the μVar variant.

Virus DNA sequencing is, however, time-consuming in the high-scale format. The identification and genotyping of highly polymorphic microsatellite areas from vertebrate herpesviruses appears as a suitable approach. Microsatellites have been reported from different herpesviruses including human cytomegalovirus and they have been used as molecular markers to define virus polymorphism [20-24].

Since the μVar variant demonstrated a deletion of 12 bp in a microsatellite locus located up-stream of the ORF4 [7], the main objective of the present study was the development of a genotyping method. This method was used to characterise 47 clinical OsHV-1 specimens by targeting this microsatellite locus. DNA sequences already available were used to compare results obtained with both techniques. Sequencing and genotyping appeared to be equally useful to differentiate clinical OsHV-1 specimens.

## Materials and methods

### Oyster samples and C2/C6 sequences

Forty-seven samples of the Pacific cupped oyster, *C. gigas,* were selected in the present study in order to analyze them by genotyping. These included animals collected from 1993 to 2010 and covered different stages of development (larvae, spat and adults) (Table 1). Most of the samples (45) were collected in France during mortality outbreaks recorded by the national network for mollusc disease monitoring (Repamo, Ifremer) and were stored frozen at −20 °C. Two samples were of different geographical origins (Japan and USA) (Table 1).

**Table 1:** **Collection of DNA extracted from *C. gigas *** **samples: geographical origins, virus DNA amounts and related GenBank accession numbers**

**Isolate code**	**Origin**	**Nature of sample**	**Viral DNA quantity (genome copies ng**^**-**^**1 of total DNA)**	**GenBank accession number**
				**C2/C6**
1993/002	France	Larvae	5.15E + 04	JN80065
1994/005	France	< 1 year	6.54E + 03	JN80068
1994/006	France	< 1 year	4.04E + 04	JN80069
1994/011	France	< 1 year	6.60E + 04	JN80070
1994/012	France	Larvae	3.79E + 05	JN80071
1995/020	France	Larvae	1.35E + 05	JN80072
2005/001	France	< 1 year	7.05E + 04	JN80081
2005/005	France	< 1 year	1.23E + 05	JN80082
2005/012	France	< 1 year	9.96E + 04	JN80084
2006/005	France	Larvae	3.21E + 03	JN80087
2006/009	France	< 1 year	2.21E + 04	JN80088
2006/018	France	< 1 year	1.31E + 04	JN80090
2007/004	France	Larvae	1.03E + 06	JN80091
2007/026	France	< 1 year	1.41E + 04	JN80094
2007/028	France	< 1 year	1.00E + 04	JN80095
2007/029	France	< 1 year	1.04E + 05	JN80096
2007/030	France	< 1 year	1.21E + 04	JN80097
2007/034	France	1-2 years	1.18E + 04	JN80098
2007/035	France	< 1 year	8.93E + 03	JN80099
2008/017	France	< 1 year	4.30E + 02	JN800100
2008/019	France	1-2 years	8.43E + 06	JN800101
2008/021	France	Larvae	2.45E + 06	JN800102
2008/023	France	Adult	2.65E + 05	JN800103
2008/020	France	Larvae	1.66E + 03	-
2008/025	France	Adult	4.33E + 05	JN800104
2008/045	France	1-2 years	8.60E + 02	JN800107
2008/050	France	< 1 year	4.68E + 02	JN800108
2008/055	France	< 1 year	2.56E + 05	JN800109
2008/059	France	< 1 year	2.60E + 05	JN800110
2008/073	France	1-2 years	4.55E + 04	JN800111
2008/079	France	< 1 year	1.45E + 04	JN800112
2008/083	France	< 1 year	2.74E + 04	JN800113
2008/092	France	< 1 year	2.24E + 03	JN800114
2009/002	France	< 1 year	2.67E + 03	JN800115
2009/021	France	< 1 year	7.17E + 03	JN800116
2009/022	France	< 1 year	1.04E + 04	JN800117
2009/027	France	< 1 year	3.31E + 03	JN800118
2009/035	France	< 1 year	2.19E + 03	JN800119
2010/002	France	< 1 year	4.44E + 04	JN800120
2010/008	France	< 1 year	2.57E + 04	JN800121
2010/012	France	< 1 year	1.58E + 05	JN800122
2010/013	France	< 1 year	1.61E + 05	JN800123
2010/021	France	< 1 year	8.38E + 03	JN800124
2010/023	France	< 1 year	6.42E + 05	JN800125
2010/026	France	< 1 year	5.98E + 05	JN800126
2007/07-CB2	USA	-	5.02E + 04	JN800128
2010/158-144	Japan	< 1 year	3.00E + 02	JN800133

Total nucleic acids were previously extracted from oyster samples using the QIAamp DNA Mini Kit (Qiagen, Courtaboeuf, France) [25] and the quantity of viral DNA was estimated by real time PCR using the primer pair C9/C10 [26] for the purpose of a previous study [10]. Sequences of C2/C6 [27] PCR products from the sample set were previously defined in the laboratory [10] and deposited in GenBank (Table 1).

### Genotyping of a microsatellite locus

A microsatellite locus (called H10) was identified up-stream of ORF4 based on an analysis of the whole genome of OsHV-1 (GenBank accession n° AY509523) using the MsatFinder algorithm [28] and selected for genotyping. The number of repeat units of this trinucleotide microsatellite was 8 in the reference sequence (GenBank accession n° AY509523). This microsatellite (H10) was located in inverted repeated regions (4440–4463 positions and 178547–178547 positions). A primer pair (H10F/H10R) was designed with primer3 [29] (H10F: gtgatggctttggtcaaggt and H10R: ggcgcgatttgtcagtttag). The expected size of the PCR product was 151 bp.

PCR was performed in 20 μL reaction volumes in a thermocycler (Applied Biosystems, Villebon-sur-Yvette, France). Each reaction contained 11.76 μL dH_2_O, 4.0 μL buffer 5X (Promega, Charbonnières-les-Bains, France), 0.8 μL MgCl_2_ (25 mM), 2 μL dNTP (2 mM), 0.18 μL of each primer (20 μM), and 0.08 μL GoTaq®DNA polymerase (5U/μL, Promega). PCR cycling conditions were 96 °C for 5 min; then 30 cycles of 95 °C for 30 s, 57 °C for 30 s and 72 °C for 30 s; and finally a step of 72 °C for 2 min. PCR products were verified by electrophoresis (1% agarose gel).

PCR products were mixed with formamide and GeneScan 500-ROX size standard (Applied Biosystems) respectively according to the manufacturer’s recommendations (1.5 μL PCR products, 0.25 μL Rox size standard and 13.25 μL formamide). After 5 min denaturation followed by rapid cooling, PCR products were detected using an ABI 3130*xl* Genetic Analyzer (Applied Biosystems), and the fragment length was estimated through the GeneMapper 3.7 software.

### Phylogenetic analysis

Phylogenetic analysis was performed on C2/C6 [27] sequences [10] using 3 computational approaches. Information concerning genotyping results (length of the fragment) was included in specimen codes. For the first approach, a phylogenetic tree was created from sequence alignments using the Neighbor-Joining (NJ) method [30]. The significance of the branching orders was assessed by bootstrap resampling of 1000 replicates. The second approach was based on phylogeny inference according to the Maximum Likelihood method based on the Tamura-Nei model [31]. Bootstrap data sets (1000 replicates) were generated. The Maximum Parsimony method was also used as the third approach. All approaches were implemented using the MEGA5 program [32].

## Results

### Polymorphism of C2/C6 PCR products

**Figure 1:**
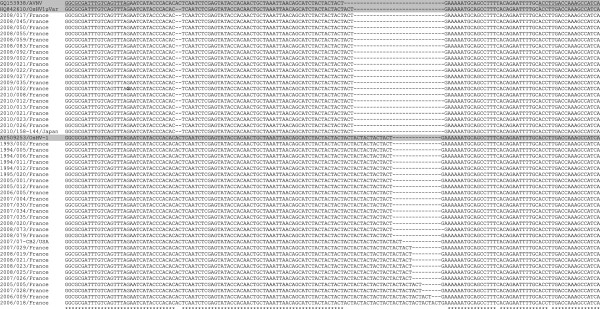
**H10F/H10R sequence alignments between virus specimens.** Partial C2-C6 (H10F/H10R) sequence alignments between virus specimens demonstrating variability at the microsatellite locus (H10) and mutation points. Locations of H10F/H10R primers are identified as surligned. OsHV-1 reference type, the variant μVar and AVNV sequences are highlited in grey. Stars represent identity at a particular nucleotide position.

Comparing sequences of C2/C6 PCR products (Table 1) demonstrated a high polymorphism with 82 positions of a 482 nucleotide sequence (17%) showing a substitution/deletion/insertion defining 9 virus specimen types from the analysed samples (data not shown). H10F/H10R sequences revealed only a variability in the number of repeat units at the targeted short tandem repeat (H10) defining 7 virus specimen types from the analysed samples (Figure 1). An additional type corresponded to acute viral necrosis virus (AVNV) infecting cultured scallops, *Chlamys farreri,* in China [33] (Figure 1). The minimum and maximum numbers of repeat units of the trinucleotide motif were 3 (AVNV) and 13 (2006/18/France), respectively (Figure 1).

### Microsatellite genotyping

The 47 samples were genotyped for the H10 microsatellite locus which is located up-stream of the ORF4. In this region, a 13 bp deletion is one of the characteristics of the μVar variant [7] and was hence chosen to achieve its interest as a diagnostic tool. Protocol optimisation focussed on the concentration of the labelled primers and the DNA in the PCR mix, but also the annealing temperature, time of elongation, and finally the dilution factor of the PCR products in the formamide before fragment length analysis in the Genetic Analyzer.

**Figure 2:**
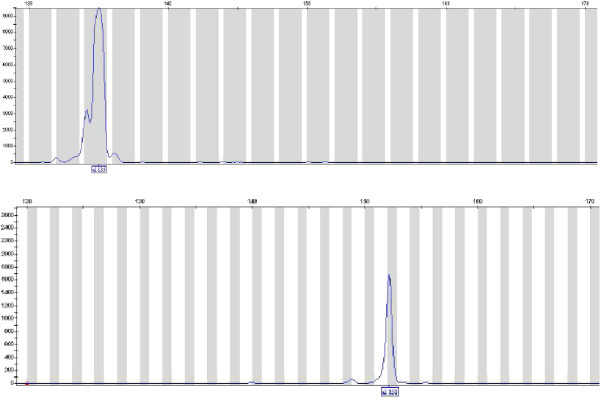
**Electrophoregrams from virus specimens.** The two traces represent separate reactions on 2 DNA extracts (2008–017 and 2008–020). The boxed numbers under the peaks of the traces are the fragment sizes in base pairs assigned by comparison with the standard curve generated with the internal size standard.

**Figure 3:**
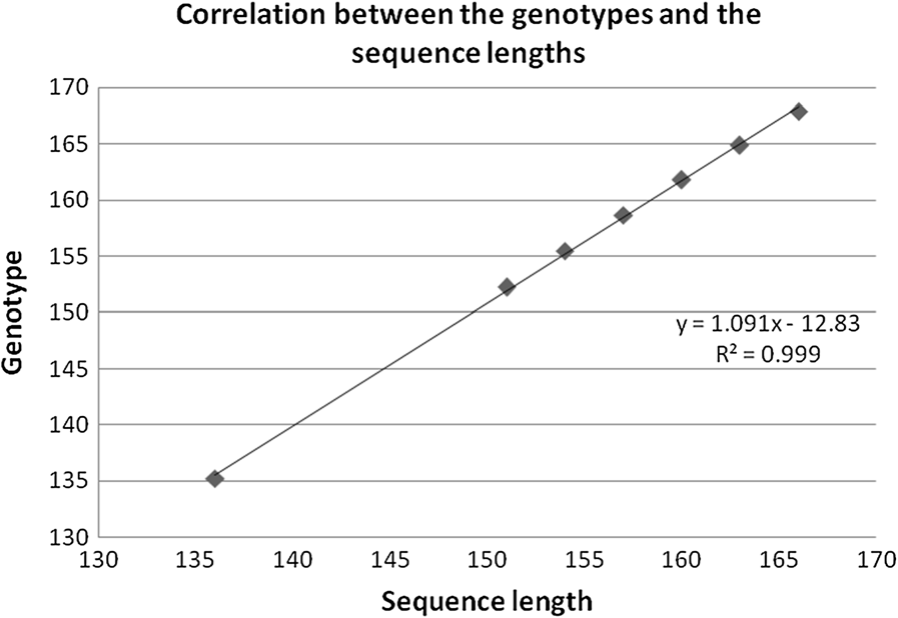
**Correlation between genotype corresponding sequence lengths.** Correlation between the genotypes detected by genotyping and the corresponding sequence lengths of the fragment obtained by sequencing for virus specimens.

Fragments were successfully amplified for the 47 samples (Figure 2). Seven different genotypes were detected corresponding to different fragment lengths estimated through the GeneMapper 3.7 software (135, 152, 155, 159, 161, 165 and 168 bp; Figure 2 and Table 2). Two genotypes (135 and 152) were more frequent with 20 and 16 of the samples, respectively (Table 2). Five more genotypes were detected in 11 samples (Table 2). When comparing the detected genotypes and the corresponding H10F/H10R sequences (Table 2), a good correlation was reported (Figure 3) showing that the genotypes identified by genotyping of the microsatellite reflected the sequences and allowed a clear discrimination between them. Moreover, sequencing showed that specimens presenting a fragment length estimated at 135 bp and 152 bp corresponded to the reference type and the μVar variant, respectively. Although all the samples collected in France in 1993, 1994 and 1995 demonstrated a fragment length estimated at 152 bp, French samples collected in 2009 and 2010 showed a single pattern at 135 bp. (Table 2). Different genotypes were detected each year for samples collected in France from 2005 to 2008 (Table 2).

**Table 2:** Results obtained for each analysed specimen in terms of genotyping and DNA sequencing were grouped per year of collection

**Specimen code**	**Genotyping**	**Number of repeats (CTA)**	**Detection frequency (%)**	**DNA sequencing**
1993/002	152,19	8	34	151
1994/005	152,19	8	34	151
1994/006	152,18	8	34	151
1994/011	152,18	8	34	151
1994/012	152,19	8	34	151
1995/020	152,16	8	34	151
2005/001	152,23	8	34	151
2005/005	161,71	11	4.3	160
2005/012	152,27	8	34	151
2006/005	152,27	8	34	151
2006/009	164,88	12	2	163
2006/018	167,91	13	2	166
2007/004	152,35	8	34	151
2007/026	158,69	10	12.7	157
2007/028	161,82	11	4.3	160
2007/029	158,67	10	12.7	157
2007/030	152,18	8	34	151
2007/034	152,32	8	34	151
2007/035	152,32	8	34	151
2008/017	135,28	4	42.5	136
2008/019	158,56	10	12.7	157
2008/020	151,9	8	34	151
2008/021	158,72	10	12.7	157
2008/023	158,68	10	12.7	157
2008/025	158,65	10	12.7	157
2008/045	135,25	4	42.5	136
2008/050	135,18	4	42.5	136
2008/055	135,3	4	42.5	136
2008/059	135,17	4	42.5	136
2008/083	135,18	4	42.5	136
2008/092	135,21	4	42.5	136
2008/073	152,3	8	34	150
2008/079	152,32	8	34	151
2009/002	135,22	4	42.5	136
2009/021	135,14	4	42.5	136
2009/022	135,28	4	42.5	136
2009/027	135,29	4	42.5	136
2009/035	135,29	4	42.5	136
2010/002	135,29	4	42.5	136
2010/008	135,23	4	42.5	136
2010/012	135,24	4	42.5	136
2010/013	135,18	4	42.5	136
2010/021	135,31	4	42.5	136
2010/023	135,17	4	42.5	136
2010/026	135,24	4	42.5	136
2007/07-CB2	155,47	9	2	154
2010/158-144	135,17	4	42.5	136

Although several genotypes were reported the same year in 2005, 2006, 2007 and 2008, a single one was noticed the other years.

### Phylogenetic analysis

**Figure 4:**
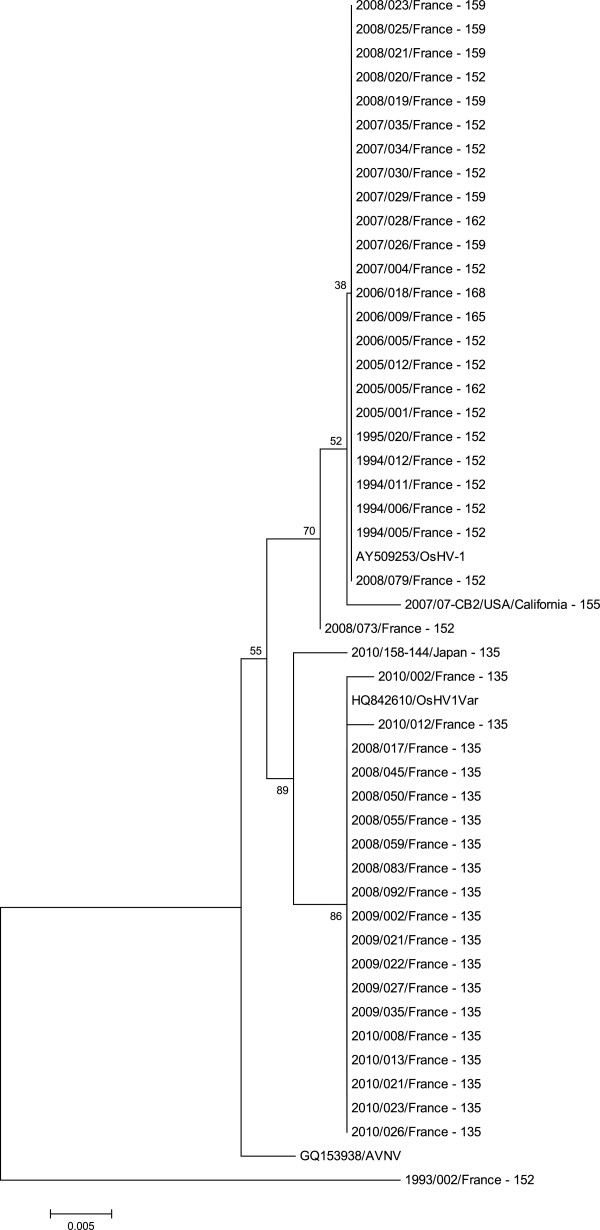
**Phylogenetic tree representing the relationships of virus specimens.** Phylogenetic tree representing the relationships of 47 virus specimens (fragment lengths obtained by genotyping were included in specimen codes) and 3 reference sequences (OsHV-1, the variant μVar and AVNV) based on a fragment of the ORF4 and its up-stream zone (460 nts). Fragment lengths were included in specimen codes. The analysis involved 50 nucleotide sequences. Evolutionary analysis was conducted in MEGA5. The tree was generated by the Maximum Likelihood method.

The phylogenetic trees built from the C2/C6 amplicon sequences (ORF4 and its related up stream area) using 3 different approaches allowed identification of 2 major groups from the 47 analysed virus specimens (Figure 4). A first group contained French specimens collected from 1993 to 2008 including the reference type (OsHV-1, GenBank n° accession AY509253) and the sample collected in the USA (California) in 2007. Although a main genotype (152 bp) was represented in this group, several other genotypes were also observed (155, 159, 162, 165 and 168 bp) (Figure 4). The second large group comprised French specimens collected from 2008 to 2010. It included the sequence of the μVar variant deposited in GenBank (accession n° HQ842610) and the sequence of the sample from Japan (Figure 4). All samples grouped with the μVar variant demonstrated a similar genotype at 135 bp (Figure 4). Two additional groups were defined. Each of them included a single member: a virus specimen collected in France in 1993 on the one hand and AVNV on the other hand.

## Discussion

This study reports for the first time the use of a microsatellite locus (H10) present in the OsHV-1 genome to analyze the virus diversity using 47 OsHV-1 specimens. Microsatellites are short tandem repeats that occur in eukaryote, prokaryote, and also some virus genomes. They are highly DNA mutable sequences and represent hot spots of length mutation. Replication slippage errors are considered as the main cause of insertions and deletions at microsatellite loci. Microsatellites have thus been extensively used as molecular markers in numerous genetic diversity and genome mapping studies. Short microsatellite polymorphisms have already been used to describe genetic polymorphism of different vertebrate herpesviruses [20-24]. As an example, Deback et al. [24] used the microsatellite technology to determine genetic relationship between HSV-1 strains and showed that each patient was characterized by its own HSV-1 microsatellite haplotype.

The microsatellite selected in the present study (H10) is found in a noncoding region. This microsatellite was selected since numerous sequences are already available for this region demonstrating a high level of length polymorphism [7,10,12,19].

In the present study analysis of C2-C6 sequences [10] was first carried out to identify polymorphisms among the selected OsHV-1 specimens and to prepare genotyping. The ORF4 area with 82 substitutions/deletions/insertions appeared highly polymorphic presenting variability in the number of repeat units at the targeted short tandem repeat and a variety of point mutations defining 9 virus types. Sequence alignment allowed identification of polymorphisms among virus specimens interpreted as being the reference type (GenBank AY509253). Several French samples collected from 1993 to 2008 demonstrated 100% identity with the reference type and as such could be identified as OsHV-1 [3]. Other samples collected in France from 2003 to 2008 showed some differences in comparison with the reference type. Finally, a French virus specimen collected in 1993 presented high homologies with the variant OsHV-1 Var [14,16,34]. These results showed that different OsHV-1 variants are represented in the sample set selected for the present study. Acute Viral Necrosis Virus (AVNV) [33] was included in comparing C2-C6 PCR product sequences sinced its complete genome is available in GenBank and it presents the shorter sequence for the H10 microsatellite. The number of sequences from countries other than France used in this study was low. Complementary analysis of additional specimens is ongoing in the laboratory and detailed comparison of sequences would present further epidemiological information on OsHV-1.

Among the 47 samples analysed, 7 different genotypes were detected with 2 more frequent ones. They respectively included specimens interpreted as the reference type and the μVar variant. Five more genotypes were also detected. When comparing the genotypes detected and the corresponding C2/C6 sequences, a good correlation was reported showing that the detected genotypes reflected the sequences and allowed a clear discrimination between specimens. However, the number of virus specimen types (9) obtained by sequencing of C2-C6 PCR products remains higher than the number of genotypes defined by genotyping (7). Although analysis of variation in length through genotyping offers a first order of discrimination, sequencing of alleles and viral length variants adds a second level. Sequencing is a necessary step to obtain maximum resolution between viral specimens also revealing SNP.

The polymorphism reported for the selected microsatellite in the present study confirms the interest of such analysis to describe OsHV-1 genome diversity. Moreover, a multiplex genotyping based on analysis of several microsatellites needs to be developed for OsHV-1. The in silico analysis of the OsHV-1 genome using the MsatFinder algorithm demonstrated the presence of 12 short repeat sites including 4 mononucleotide units, 5 dinucleotide repeats, and 3 trinucleotide repeats (data not shown). Most of the identified microsatellites were localized in noncoding parts of the OsHV-1 genome, except for 3 of them located in ORF 66, 77 and 106, respectively. The number of repeat units of dinucleotide or trinucleotide microsatellites was 5 or 8. The longest mononucleotide sequence was 18 bases and 3 microsatellites were located in inverted repeated regions. As most of OsHV-1 repeats are found in noncoding areas, they can be considered as evolutionarily neutral or nearly so and therefore as suitable markers for epidemiology studies. Such a technique targeting several microsatellite loci may provide a rapid and accurate tool that can be used to compare OsHV-1 specimens and to study the epidemiology of viral infections. Finally, polymorphism of microsatellites may also be used to study viral strain virulence. Perdue et al. [35] reported that the increased virulence of a particular strain of the avian influenzae virus is related to the increase in the length of a microsatellite at the hemagglutinin cleavage site.

In conclusion, genotyping based on microsatellite loci appears as a powerful tool to study OsHV-1 polymorphism and can offer a first level of discrimination between specimens in order to select best candidates for complete genome sequencing. Futhermore comparative diversity studies between the host, *Crassostrea gigas*, and OsHV-1 can be easily performed using oyster mircosatellite markers [36] and to characterize coevolution in this recently introduced oyster species in Europe [37].

## Competing interests

This work was supported by Ifremer (Institut Français pour l’Exploitation de la Mer). The authors declare that they have no competing interests.

## Authors’ contributions

This study is the result of a collective work. TR and SL conceived this study, and participated in its design. TR, GT and SL carried out the design of the protocol, with the help of the other authors. GT, NF, PM and AS carried out sample analyses. TR and SL drafted the manuscript. VBS participated in the discussion and modification of the manuscript. All authors read, corrected, and approved the final manuscript.

## References

[B1] Davison AJ Eberle R Ehlers B Hayard GS McGeoch DJ Minson AM Pellett PE Roizman B Studdert MJ Thiry E The order *Herpesvirales* Arch Virol 2009 154 171 177 10.1007/s00705-008-0278-4 19066710 PMC3552636

[B2] Le Deuff RM Renault T Purification and partial genome characterization of a herpes-like virus infecting the Japanese oyster, *Crassostrea gigas* J Gen Virol 1999 80 1317 1322 10355779 10.1099/0022-1317-80-5-1317

[B3] Davison AJ Trus BL Cheng N Steven AC Watson MS Cunningham C Le Deuff RM Renault T A novel class of herpesvirus with bivalve hosts J Gen Virol 2005 86 41 53 10.1099/vir.0.80382-0 15604430

[B4] Renault T Le Deuff RM Cochennec N Maffart P Herpesviruses associated with mortalities among Pacific oyster, *Crassostrea gigas*, in France - Comparative study Rev Med Vet 1994 145 735 742

[B5] Garcia C Thébault A Dégremont L Arzul I Miossec L Robert M Chollet B François C Joly JP Ferrand S Kerdudou N Renault T OsHV-1 detection and relationship with *C. gigas* spat mortality in France between 1998 and 2006 Vet Res 2011 42 73 84 10.1186/1297-9716-42-73 21635731 PMC3129302

[B6] Efsa Scientific opinion on the increased mortality events in Pacific oysters, *Crassostrea gigas* EFSA J 2010 8 1894 1954

[B7] Segarra A Pépin JF Arzul I Morga B Faury N Renault T Detection and description of a particular *Ostreid herpesvirus 1* genotype associated with massive mortality outbreaks of Pacific oysters, *Crassostrea gigas* Virus Res 2010 153 92 95 10.1016/j.virusres.2010.07.011 20638433

[B8] Lynch SA Carlson J Reilly AO Cotter E Culloty SC A previously undescribed ostreid herpes virus 1 (OsHV-1) genotype detected in the Pacific oyster, *Crassostrea gigas*, in Ireland Parasitology 2012 139 1526 1532 10.1017/S0031182012000881 23036593

[B9] Peeler JE Reese RA Cheslett DL Geoghegan F Power A Trush MA Investigation of mortality in Pacific oysters associated with Ostreid herpesvirus-1 μVar in the Republic od Ireland in 2009 Prev Vet Med 2012 105 136 143 10.1016/j.prevetmed.2012.02.001 22398251

[B10] Renault T Moreau P Faury N Pépin JF Segarra A Webb S Analysis of clinical ostreid herpesvirus 1 (*Malacoherpesviridae*) specimens by sequencing amplified fragments from three virus genome areas J Virol 2012 86 5942 5947 10.1128/JVI.06534-11 22419803 PMC3347280

[B11] Roque A Carrasco N Andree KB Lacuesta B Elandaloussi L Gairin I Rodgers CJ Furones MD First report of OsHV-1 microvar in Pacific oyster (*Crassostrea gigas*) cultured in Spain Aquaculture 2012 324 303 306

[B12] Martenot C Oden E Travaillé E Malas JP Houssin M Detection of different variants of Ostreid Herpesvirus 1 in the Pacific oyster *Crassostrea gigas* Virus Res 2011 160 25 31 10.1016/j.virusres.2011.04.012 21600247

[B13] Norberg P Bergström T Liljeqvist JA Genotyping of clinical Herpes Simplex Virus type 1 isolates by use of restriction enzymes J Clin Microbiol 2006 44 4511 4514 10.1128/JCM.00421-06 17035491 PMC1698414

[B14] Arzul I Nicolas JL Davison AJ Renault T French scallops: a new host for ostreid herpesvirus 1 Virology 2001 290 342 349 10.1006/viro.2001.1186 11883198

[B15] Arzul I Renault T Lipart C Davison AJ Evidence for interspecies transmission of oyster herpesvirus in marine bivalves J Gen Virol 2001 82 865 870 11257192 10.1099/0022-1317-82-4-865

[B16] Renault T Lipart C Arzul I A herpes-like virus infecting *Crassostrea gigas* and *Ruditapes philippinarum* larvae in France J Fish Dis 2001 24 369 376 10.1046/j.1365-2761.2001.00300.x 11411639

[B17] Friedman CS Estes RM Stokes NA Burge CA Hargove JS Barber BJ Elston RA Burreson EM Reece KS Herpes virus in juvenile Pacific oysters Crassostrea gigas from Tomales Bay, California, coincides with summer mortality episodes Dis Aquat Organ 2005 63 33 41 15759798 10.3354/dao063033

[B18] Moss JA Burreson EM Cordes JF Dungan CF Brown GD Wang A Wu X Reece KS Pathogens in *Crassostrea ariakensis* and other Asian oyster species: implications for non-native oyster introduction in Chesapeake Bay Dis Aquat Organ 2007 77 207 233 18062472 10.3354/dao01829

[B19] Shimahara Y Kurita J Kiryu I Nishioka T Yuasa K Kawana M Kamaishi T Oseko N Surveillance of Type 1 Ostreid Herpesvirus (OsHV-1) variants in Japan Fish Pathol 2012 47 129 136 10.3147/jsfp.47.129

[B20] Davis CL Field D Metzgar D Saiz R Morin PA Smith IL Spector SA Wills C Numerous length polymorphism at short tandem repeats in human cytomegalovirus J Virol 1999 73 6265 6270 10400717 10.1128/jvi.73.8.6265-6270.1999 PMC112704

[B21] Walker A Petheram SJ Ballard L Murph JR Demmler GJ Bale JF Characterization of human cytomegalovirus strains by analysis of short tandem repeat polymorphisms J Clin Microbiol 2001 39 2219 2226 10.1128/JCM.39.6.2219-2226.2001 11376060 PMC88114

[B22] Picone O Costa JM Ville Y Rouzioux C Leruez-Ville M Human cytomegalovirus (HCMV) short tandem repeats analysis in congenital infection J Clin Virol 2005 32 254 256 10.1016/j.jcv.2004.10.012 15722033

[B23] Deback C Boutolleau D Depienne C Luyt CE Bonnafous P Gautheret-Dejean A Garrigue I Agut H Utilization of microsatellite polymorphism for differentiating herpes simplex virus type 1 strains J Clin Microbiol 2008 47 533 540 19109460 10.1128/JCM.01565-08 PMC2650923

[B24] Deback C Luyt CE Lespinats S Depienne C Boutolleau D Chastre J Agut H Microsatellite analysis of HSV-1 isolates: from oropharynx reactivation toward lung infection in patients undergoing mechanical vantilation J Clin Virol 2010 47 313 320 10.1016/j.jcv.2010.01.019 20172760

[B25] Qiagen QIAmp® DNA Mini and Blood Mini Handbook 2007 2 D-46724 Hilden, Germany: QIAGEN Gmbh

[B26] Pépin JF Riou A Renault T Rapid and sensitive detection of ostreid herpesvirus A in oysters samples by real-time PCR J Virol Methods 2008 149 269 276 10.1016/j.jviromet.2008.01.022 18342377

[B27] Renault T Arzul I Herpes-like virus infections in hatchery-reared bivalve larvae in Europe: specific viral DNA detection by PCR J Fish Dis 2001 24 161 167 10.1046/j.1365-2761.2001.00282.x

[B28] Thurston MI Field D Msatfinder: detection and characterisation of microsatellites [http://www.bioinformatics.org/ftp/pub/msatfinder/README.txt]

[B29] Rozen S Skaletsky H Krawetz S, Misener S Primer3 on the WWW for general users and for biologist programmers Bioinformatics Methods and Protocols: Methods in Molecular Biology 2000 Totowa, NJ: Humana Press 365 386 10.1385/1-59259-192-2:365 10547847

[B30] Saitou N Nei M The neighbor-joining method: a new method for reconstructing phylogenetic trees Mol Biol Evol 1987 4 406 425 3447015 10.1093/oxfordjournals.molbev.a040454

[B31] Tamura K Peterson D Peterson N Stecher G Nei M Kumar S MEGA5: molecular evolutionary genetics analysis using maximum likelihood, evolutionary distance, and maximum parsimony methods Mol Biol Evol 2011 28 2731 2739 10.1093/molbev/msr121 21546353 PMC3203626

[B32] Tamura KM Nei M Estimation of the number substitutions in the control region of mitochondrial DNA in humans and chimpanzees Mol Biol Evol 1993 10 512 526 8336541 10.1093/oxfordjournals.molbev.a040023

[B33] Ren W Chen H Renault T Cai Y Bai C Wang C Huang J Complete genome sequence of acute viral necrosis virus associated with massive mortality outbreaks in the Chinese scallop, *Chlamys farreri* Virol J 2013 10 110 116 10.1186/1743-422X-10-110 23566284 PMC3623871

[B34] Renault T Lipart C Arzul I A herpes-like virus infects a non-ostreid bivalve species: virus replicaton in *Ruditapes philippinarum* larvae Dis Aqua Organ 2001 45 1 7 10.3354/dao045001 11411639

[B35] Perdue ML Garcia D Senne D Fraire M Virulence-associated sequence duplication at the hemagglutinin cleavage site of avian influenza viruses Virus Res 1997 49 173 186 10.1016/S0168-1702(97)01468-8 9213392

[B36] Li R Li Q Cornette C Dégremont L Lapègue S Development of four EST-SSR multiplex PCRs in the Pacific oyster *(Crassostrea gigas*) and their validation in parentage assignment Aquaculture 2010 310 234 239 10.1016/j.aquaculture.2010.09.037

[B37] Rohfritsch A Bierne N Boudry P Heurtebise S Cornette F Lapègue S Population genomics shed light on the demographic and adaptive histories of European invasion in the Pacific oyster, *Crassostrea gigas* Evol Appl 2013 6 1064 1078 24187588 10.1111/eva.12086 PMC3804239

